# American Meat Science Association News

**DOI:** 10.1093/af/vfz032

**Published:** 2019-09-28

The American Meat Science Association (AMSA) fosters community and professional development among individuals who create and apply science to efficiently provide safe and high quality meat.

## AMSA Names Industry Veteran, Collette Kaster as the New AMSA CEO


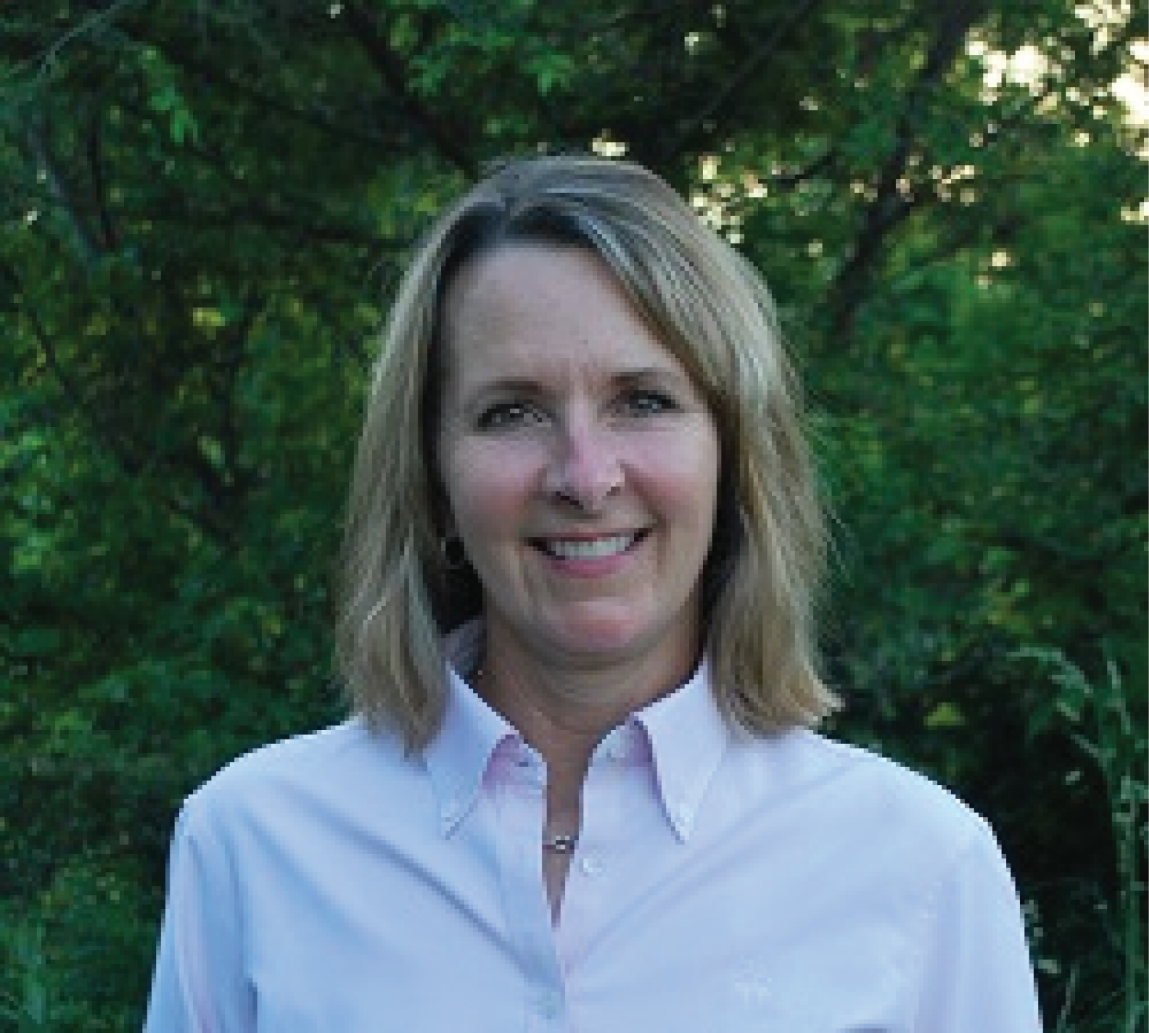


AMSA announced the appointment of Collette Schultz Kaster as its new Chief Executive Officer on June 6, 2019. Kaster brings more than 25 years of technical experience in areas of food safety, quality assurance, and animal welfare and executive roles managing teams across livestock industry companies and organizations. Most recently, she has served as the Executive Director of the Professional Animal Auditor Certification Organization (PAACO).

“Collette is the right leader at the right time for AMSA and its future. She has extensive industry knowledge combined with her formidable experience managing successful teams and businesses. She understands our organization and has a vision to meet member needs in this growing association and provide content and context for the science and information demands of an expanding world,” stated Dr. Eric Berg, AMSA President.

Kaster is trained as a meat scientist, with degrees from South Dakota State University and the University of Nebraska. She has been a member of AMSA for 28 years and is a past President and holds the rank of AMSA Fellow. In addition to her new role with AMSA, Kaster will continue to lead PAACO as its Executive Director. To enable this dual role, PAACO has developed several highly experienced Training Specialists to meet the growing needs for animal auditor training, certification, and audit certification across all sectors of the livestock industry. Kaster began as the CEO for AMSA on July 1.

### 2019 Calendar of Events:

October 13 - American Royal Meat Judging Contest - Omaha, Nebraska

October 15 – National 4H Meat Judging and Identification Contest – Manhattan, Kansas

October 21–23- PORK 101 - Iowa State University - Ames, Iowa

October 27 - Cargill High Plains Meat Judging Contest - Friona, Texas

November 10 - International Intercollegiate Meat Judging Contest - Dakota City, Nebraska

### Reciprocal Meat Conference 2019–2020

June 23–26, 2019 - Colorado State University - Fort Collins, Colorado

August 2–7, 2020 – RMC and ICoMST - Disney Coronado Springs Resort in Lake Buena Vista, Florida, USA

